# Metal-Formate Framework Stiffening and Its Relevance to Phase Transition Mechanism

**DOI:** 10.3390/ma14206150

**Published:** 2021-10-16

**Authors:** Paulina Peksa, Justyna Trzmiel, Maciej Ptak, Aneta Ciupa-Litwa, Adam Sieradzki

**Affiliations:** 1 Department of Experimental Physics, Wrocław University of Science and Technology, Wybrzeże Wyspiańskiego 27, 50-370 Wrocław, Poland; paulina.peksa@pwr.edu.pl; 2 Department of Theoretical Physics, Wrocław University of Science and Technology, Wybrzeże Wyspiańskiego 27, 50-370 Wrocław, Poland; justyna.trzmiel@pwr.edu.pl; 3 Institute of Low Temperature and Structure Research, Polish Academy of Sciences, Box 1410, 50-950 Wrocław, Poland; m.ptak@intibs.pl (M.P.); a.ciupa@intibs.pl (A.C.-L.)

**Keywords:** organic-inorganic hybrids, metal-formate frameworks, dielectric spectroscopy

## Abstract

In the last decade, one of the most widely examined compounds of motal-organic frameworks was undoubtedly ((CH_3_)_2_NH_2_)(Zn(HCOO)_3_), but the problem of the importance of framework dynamics in the order–disorder phase change of the mechanism has not been fully clarified. In this study, a combination of temperature-dependent dielectric, calorimetric, IR, and Raman measurements was used to study the impact of ((CH_3_)_2_NH_2_)(Zn(DCOO)_3_) formate deuteration on the phase transition mechanism in this compound. This deuteration led to the stiffening of the metal-formate framework, which in turn caused an increase in the phase transition temperature by about 5 K. Interestingly, the energetic ordering of DMA^+^ cations remained unchanged compared to the non-deuterated compound.

## 1. Introduction

Recently, much research has focused on the exploration of metal-formate compounds, one of the families of metal-organic frameworks (MOF). MOFs have received a great deal of attention due to their potential applications as catalysts, biomedical sensors, and luminescent materials [1,2,3,4,5]. Most efforts have been devoted to dimethylammonium MOFs with the general formula ((CH_3_)_2_NH_2_)(M(HCOO)_3_) (DMAM, where M denotes divalent metal ions) [6,7,8,9,10,11,12,13,14,15,16,17,18,19,20]. These compounds consist of metal centers M^2+^ related by HCOO^−^ anionic ligands within the frameworks with pseudo-cubic nanocavities [15,21]. Each of these cavities contains a (CH_3_)_2_NH_2_ (DMA) cation, which organizes the H-bonds within the metal-formate framework [22,23]. 

The most studied compound in this family is dimethylamonium zinc formate [24,25]. It undergoes an order–disorder phase transition at T_c_~156 K [26,27,28,29]. In the high-temperature phase (HT), the H-bonding connection between the DMA^+^ cation and the framework is weak, so the thermal energy is strong enough to break off the H-bonds. This behavior indicates that DMA^+^ is dynamically disordered, i.e., it flips (rotates 120 degrees) between three equivalent positions, consistent with the space group R-3c [30]. At temperatures below the phase transition temperature, cations freeze in one of the favorable positions; this is indicated by the transition to the Cc phase [7,22,31]. It has previously been reported that the phase transition is driven by ordering the DMA^+^ cations. It is worth mentioning, however, that other studies, such as the neutron diffraction [32] or Raman spectroscopy [13], suggest that the phase transition in this material is related to framework deformation and intermolecular forces, especially the strength of H-bonds between the organic cation and the framework [33].

As the temperature decreases from the HT phase, near the phase transition temperature, the intermolecular forces are changed and the N^…^O bonds shorten. As a result, the metal-formate framework contracts (deforms). This slight deformation of the framework is highly likely to be the reason for the ordering of the DMA^+^ cations. The results of inverse Monte Carlo modelling performed for the ((CD_3_)_2_ND_2_)(Mn(DCO_2_)_3_) compound confirmed that near the phase transition temperature, the metal-formate framework becomes contracted, thereby inducing DMA^+^ cation ordering [32].

There have been some studies of the replacement of hydrogen with deuterium in metal-formate frameworks, as well as by those with the DMA^+^ cation [13,34], just to mention compounds with deuterated cations, such as ((CH_3_)_2_ND_2_)(M(HCOO)_3_) (M = Ni, Mn), or fully deuterated formates, such as ((CD_3_)_2_ND_2_)(Co(DCOO)_3_). The results obtained for ((CD_3_)_2_ND_2_)(Co(DCOO)_3_) and ((CH_3_)_2_ND_2_)(M(HCOO)_3_) (M = Ni, Mn) showed that in these compounds, the phase transition is driven by the ordering of the DMA^+^ cations. For these materials, it has been suggested that the observed phase transition may also be accompanied by the metal-formate framework deformation [13,34]. Similarly, it was suggested for the ((CH_3_)_2_NH_2_)(Zn(HCOO)_3_) (DMAZnF) compound that the phase transition in this material was not only related to the ordering of the DMA^+^ cations but also to the framework deformation [21]. Nevertheless, there are currently no results that clearly show the influence of the framework deuteration on the mechanism of phase transition for dimethylamonium zinc formate (DMAZnF).

The aim of this study was to verify whether the framework’s stiffening (as a consequence of its deuteration) affects the dynamics of the built-in cation movements in ((CH_3_)_2_NH_2_)(Zn(DCOO)_3_) (DMAZnD), and thus the phase stability. For this purpose, a combination of temperature-dependent dielectric, calorimetric, IR, and Raman experiments was applied. The deuteration led to the stiffening of the metal-formate framework.

## 2. Materials and Methods

Sample preparation. All the reagents, i.e., ZnCl_2_ (99.999%, Sigma-Aldrich, Saint Louis, MO, USA), a 2.0 M solution of dimethylamine in methanol, methanol (99.8%, Sigma-Aldrich, Saint Louis, MO, USA), formic acid (98–100%, POCH, Gliwice, Poland), formic-d acid (DCOOH, 95 wt.% in H_2_O, 98 atom.% D, Sigma-Aldrich, Saint Louis, MO, USA), and N,N-dimethylformamide (99.8%, Sigma-Aldrich, Saint Louis, MO, USA), were purchased and used without further purification.

In order to obtain ((CH_3_)_2_NH_2_)Zn(DCOO)_3_ (DMAZnD) and ((CH_3_)_2_NH_2_)Zn(HCOO)_3_ (DMAZnF), 2 mL of a 2.0 M solution of dimethylamine in methanol, 1 mL of formic-d acid for DMAZnD or formic acid for DMAZnF, and 10 mL of N,N-dimethylformamide were added to 10 mL of methanol and mixed. In the next step, 10 mL of methanol solution containing 1 mmol of ZnCl_2_ were added, mixed, and left at room temperature (RT) in a sealed polypropylene container. After 48 h, the crystals were harvested, washed three times with methanol, and dried at RT.

X-ray diffraction. The powder XRD (X-ray diffraction) patterns were obtained on an X’Pert PRO XRD (Malvern Panalytical Ltd., Malvern, UK) system equipped with a PIXcel ultrafast line detector, a focusing mirror, and Soller slits for Cu Kα radiation (λ = 1.54056 Å).

Thermal properties. Differential scanning calorimetry (DSC) measurements were performed using a Mettler Toledo DSC-1 calorimeter (Mettel Toledo, Zurich, Switzerland) with a scanning rate of 5 K/min on cooling/heating. The excess heat capacity associated with the phase transition was evaluated by subtraction from the data the baseline representing variation in the absence of the phase transitions.

Raman and IR spectroscopies. An RT Raman spectrum of polycrystalline DMAZnD was measured in the 3500–50 cm^−1^ range using a Bruker FT 100/S spectrometer (Bruker, Billerica, MA, USA) with a YAG:Nd laser and a excitation of 1064 nm. The temperature-dependent (80–300 K) Raman spectra of a randomly oriented single crystal of DMAZnD in the 3500–50 cm^−1^ range were measured using a Renishaw inVia Raman spectrometer (Renishaw, Wotton-under-Edge, UK), equipped with a confocal DM2500 Leica optical microscope, a thermoelectrically cooled CCD as a detector, and an Ar^+^ ion laser operating at 488 nm. The temperature was controlled using a Linkam THMS600 stage (Linkam Scientific Instruments Ltd., Epsom, Tadworth, UK) equipped with quartz windows.

A RT polycrystalline infrared (IR) spectrum of DMAZnD in the range of 4000–650 cm^−1^ was measured using a Nicolet iS50 IR spectrometer (Thermo Fisher Scientific, Waltham, MA, USA) in a KBr pellet. The temperature-dependent (80–300 K) IR spectra of the DMAZnD in the 2900–650 cm^−1^ range were measured using a Nicolet iN10 IR microscope(Thermo Fisher Scientific, Waltham, MA, USA). The temperature was controlled using a Linkam THMS600 stage (Linkam Scientific Instruments Ltd., Epsom, Tadworth, UK) equipped with ZnSe windows.

Dielectric properties. The dielectric measurements at ambient pressure were performed using a Novocontrol Alpha impedance analyzer (Novocontrol Technologies GmbH &Co. KG, Montabaur, Germany). Silver paste was used to ensure good electrical contact. An AC voltage with an amplitude of 1V and a frequency in the range of 1–10^6^ Hz was applied across the sample. The temperature was controlled by the Novocontrol Quattro system, by using a nitrogen gas cryostat (Novocontrol Technologies GmbH &Co. KG, Montabaur, Germany). All the dielectric measurements were taken on the pellet and measured every 2 K over a temperature range from 130 K to 370 K.

## 3. Results and Discussion

### 3.1. X-ray Diffraction

**Figure 1:**
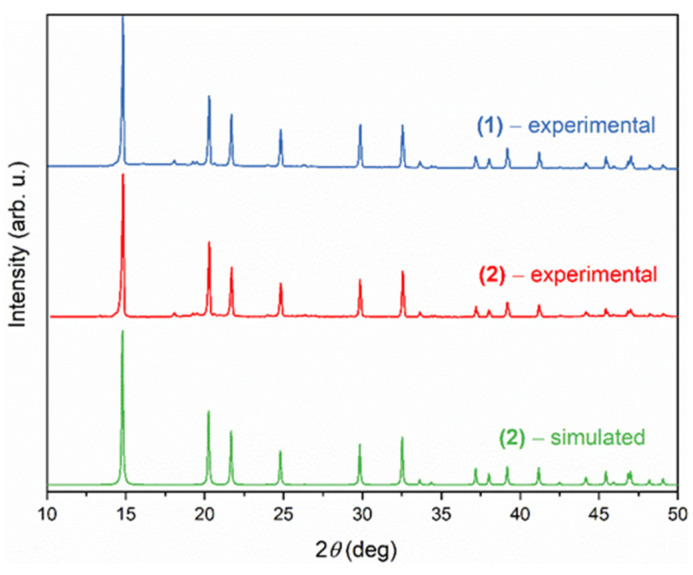
Experimental powder XRD patterns for DMAZnD (blue line) and DMAZnF (red line) compared to simulation, based on structural data presented for DMAZnF taken from [22].

To confirm the phase purity of the DMAZnD and DMAZnF powder, XRD pattern measurements were taken. The measurement was confirmed by the good agreement between the experimental powder XRD patterns compared to the simulated pattern taken from [22] (Figure 1).

### 3.2. Thermal Properties

**Figure 2:**
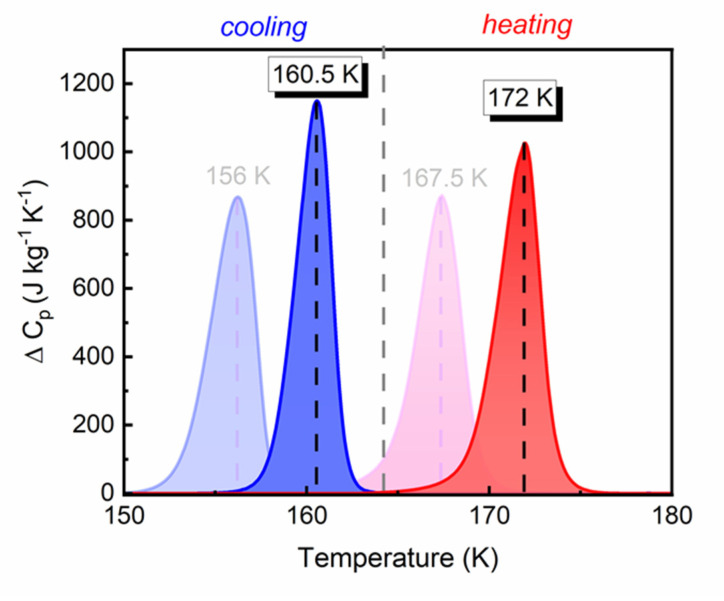
The changes in heat capacity to the phase transition in DMAZnD and DMAZnF (light background) [26] measured in cooling and heating modes.

To understand the origin of the phase transition in DMA^+^ formate frameworks, the results of the thermal measurement obtained from the DMAZnD sample were analyzed. The results obtained are shown in Figure S1. Changes in the heat capacity related to the structural phase transition in the DMAZnD and DMAZnF compounds are presented in Figure 2 Compared to the DMAZnF [26], the phase transition temperature of the DMAZnD increased to 160.5 K (156 K) upon cooling and 172 K (167.5 K) upon heating. The symmetrical shape of the heat anomaly in the DMAZnD was very similar to that observed for DMAZnF and indicated a first-order phase transition. The change in entropy ΔS associated to the phase transitions was ~4 J mol^−1^ K^−1^, suggesting the invariant mechanisms of the phase transitions in DMAZnF and DMAZnD. The estimated value of ΔS remained in good agreement with the previously reported results for DMAZnF [26]. The above results allow to conclude that the framework deuteration has an impact on the phase transition temperature DMAZnF.

Taking into account that deuteration results in the framework stiffening by increasing the strength of the interaction between hydrogen and carbon in the ligand, it can be concluded that the increase in the phase transition temperature is related to the fact that more thermal energy must be supplied to deform the framework (deformation denotes the shortening of N^...^O bonds at switching from the high- to the low-temperature phase).

### 3.3. Raman and IR Spectroscopies

To understand the vibrational properties of the DMAZnD and the DMAZnF, the labels of normal vibrations must be briefly explained. The formate ion has six fundamental vibrations: the CH stretching (ν_1_), the symmetric OCO stretching (ν_2_), the OCO bending (scissoring) (ν_3_), the antisymmetric OCO stretching (ν_4_), the in-plane CH bending (ν_5_), and the out-of-plane CH bending (ν_6_) mode. The internal vibrations of the DMA^+^ cation can be subdivided into the symmetric and antisymmetric stretching (ν_s_, ν_as_), bending (δ_s_, δ_as_), rocking (ρ), wagging (ω), and twisting (τ) vibrations of the methyl groups, the bending, wagging and twisting vibrations of the protonated amino group, as well as the stretching and bending vibrations of the CNC skeleton (see Table S1). The factor group analysis, discussed previously in detail for the DMAZnF, showed that in both the LT and HT phases the expected number of internal and lattice vibrations remained the same for both compounds [35].

The IR and Raman spectra of the DMAZnD compared to the DMAZnF are presented in Figure S2. Table S1 lists the positions of the observed IR and Raman bands and the proposed assignments based on previous vibrational studies of DMAZnF [35]. The selective deuteration of the formate ligand resulted in strong downshifts of the ν_1_–ν_6_ IR and Raman bands compared to the DMAZnF. The most pronounced downshifts (by 683–721 cm^−1^) were observed for the ν_1_ modes. The remaining characteristic vibrations of the ligand were less sensitive: the ν_2_, ν_3_, ν_4_, ν_5_, and ν_6_ modes were shifted towards lower wavenumbers by 7–67 cm^−1^. The noticeable changes in the mutual intensity of the bands accompanying deuteration were due to slight changes in the polarities of the CH and CD bonds. Small differences in the electronegativity of hydrogen isotopes affected bond lengths and charge distribution. As a result, the values of the dipole moments and the components of the polarizability tensor, which were proportional to the intensities of the IR and Raman bands, respectively, were different.

As expected, the deuteration of formate ion had a weaker effect on the positions of the bands corresponding to the DMA^+^ cations. The bands originating from the vibrations of the methyl groups and the CNC skeleton experienced shifts not exceeding 2 cm^−1^. The bands relating to H-bonds (HBs) were slightly more shifted. The IR spectra showed that the bands originating from the νNH_2_ and δNH_2_ were observed at about 5 cm^−1^ lower wavenumbers for the DMAZnD. This indicates that HBs, in which deuterium is involved, are slightly stronger compared to protium.

The deuteration effect was also evident for the Raman bands originating from the lattice vibrations, especially for those observed below 250 cm^−1^, as they received a strong contribution from the translations and vibrations of the formate ions.

**Figure 3:**
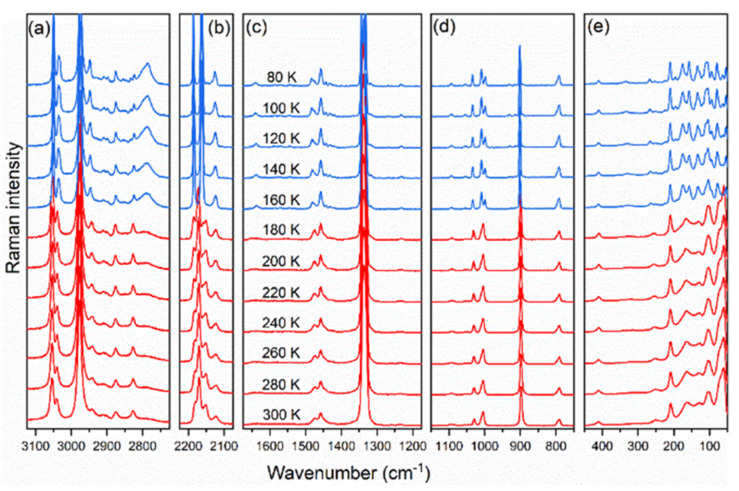
Thermal evolution of Raman spectra measured for DMAZnD at selected temperatures in a spectral range: (**a**) 3150 − 2750 cm^−1^, (**b**) 2250 − 2050 cm^−1^, (**c**) 1650 − 1150 cm^−1^, (**d**) 1150 − 750 cm^−1^, (e) 450 − 50 cm^−1^.

To shed more light on the mechanism of the phase transition and to elucidate the exact cause of the phase transition temperature increase for the DMAZnD, temperature-dependent Raman and IR spectra were measured. They are presented in Figure 3 and Figure S3, respectively. The evolution of the Raman and IR spectra on decreasing temperature measured for the DMAZnD was very similar to that observed previously for DMAZnF [35]. In particular, sudden changes resulting from the phase transition taking place at T_c_ (see the spectra below 160 K in Figure 3 and Figure S4), such as shifts, splitting, narrowing, and increase of intensity of the bands, were clearly seen. All these effects were stronger for the bands originating from the organic cation, confirming the order–disorder nature of the phase transition. The appearance of additional bands in the low-temperature (LT) phase was consistent with a reduction in symmetry from trigonal (space group R-3c) to monoclinic (space group Cc), as shown previously in the correlation diagram for DMAZnF [35].

**Figure 4:**
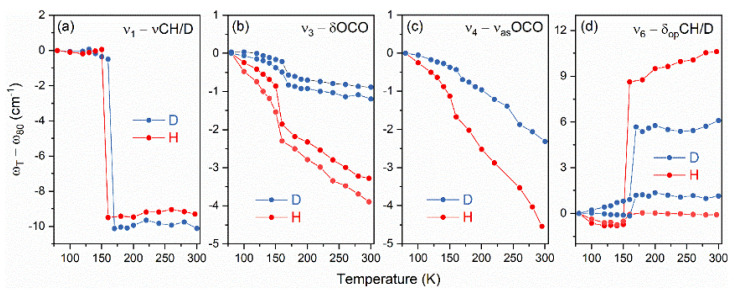
The temperature dependencies of ν_1_ (**a**), ν_3_ (**b**), ν_4_ (**c**), and ν_6_ (**d**) bands observed for deuterated (D, blue) DMAZnD and non-deuterated (H, red) analogues [35]; (**a**,**b**,**d**) presents Raman bands and (**c**) presents IR bands.

Figure 4 presents a comparison of the temperature dependencies of the band positions obtained for the DMAZnD and the DMAZnF [35], assigned to the formate ligand. Figure 4a demonstrates that the magnitude of the ν_1_ mode shifts at T_c_ did not change after deuteration. In contrast to the ν_1_ mode, the ν_3_, ν_4_ and ν_6_ modes showed some sensitivity to the deuteration (Figure 4b–d); that is, the jumps at T_c_ were almost twice as small for DMAZnD. This behaviour suggested that distortion of the framework in the LT phase decreased after deuteration. Considering slightly more robust HBs in DMAZnD, it can be inferred that the zinc-formate framework of DMAZnD is slightly stiffer compared to that of DMAZnF.

The less flexible framework of DMAZnD was expected to affect the geometry of the DMA^+^ cation and its fit to the size of the available crystal lattice voids. Figure S4 shows the temperature changes in the positions of selected IR and Raman bands assigned to the vibrations of the CNC skeleton and the methyl groups. It is clear that, as in the case of the formate ions, the νCNC stretching vibrations were less sensitive to the deuteration than the δCNC bending vibrations (Figure S4a,b). However, unlike the formate ions, the jump in wavenumber for δCNC at T_c_ was stronger for the DMAZnD compared to the DMAZnF. This is an indication that the phase transition led to larger change in the CNC angle in the DMAZnD compared to the DMAZnF. The δ_as_CH_3_ vibrations were insensitive to the deuteration, in line with the fact that they did not form HBs with the framework (see Figure S4c,d).

**Figure 5:**
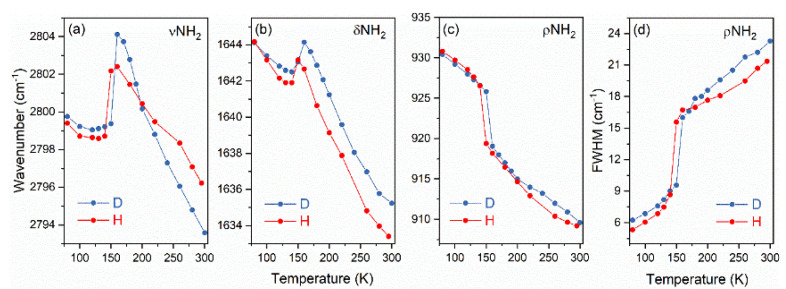
A comparison of the temperature dependencies of IR bands, corresponding to νNH_2_ (**a**), δNH_2_ (**b**), and ρNH_2_ (**c**,**d**) for DMAZnD (D, blue) and DMAZnF (H, red) [35]; the lines are guides for eyes, fwhm, full−width at half maximum.

Since the phase transition was of the order–disorder type, i.e., it was related to freezing of the thermally-induced reorientational motions of the confined DMA^+^ cation in the LT phase, the microscopic mechanism of the phase transition was likewise expected to be manifested in the evolution of the bands originating from the protonated amine group. Figure 5 confirms this assumption, since it shows large shifts and narrowing of the bands. In particular, Figure 5a shows that the νNH_2_ and δNH_2_ modes shifted to lower wavenumbers at T_c_, indicating an increase in the HBs’ strength in the LT phase. In is worth noting that all the bands corresponding to the amino group showed very similar levels of temperature dependence. This behaviour indicates that the deuteration has a very weak effect on the ordering/disordering process of DMA^+^.

### 3.4. Dielectric Studies

**Figure 6:**
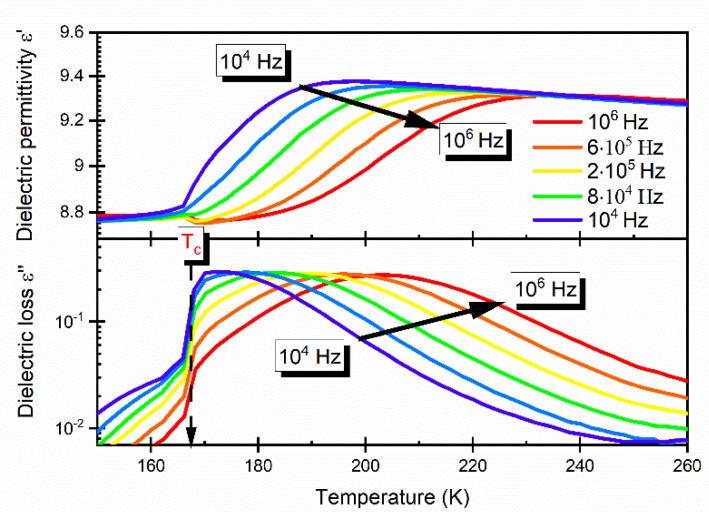
Temperature dependence of dielectric permittivity and dielectric loss measured for selected frequencies for DMAZnD.

In order to obtain an insight into the phase transition mechanism in the studied DMAZnD, dielectric spectroscopy measurements were taken. It is clear from the data presented in Figure 6 (both the real ε’ and imaginary ε’’ part of complex dielectric permittivity ε* = ε’ − iε’’) that for the studied sample, a strong dispersion was observed in the HT phase. This result indicates the presence of a thermally activated relaxation process. The bell shape of ε’’ suggests the dipolar relaxation response of the material. The observed discontinuity of both ε’ and ε’’ datasets at T_0_~170 K indicates the first-order structural phase transition, which is in good agreement with the results of the other studies performed on DMAZnF [24,30,34].

**Figure 7:**
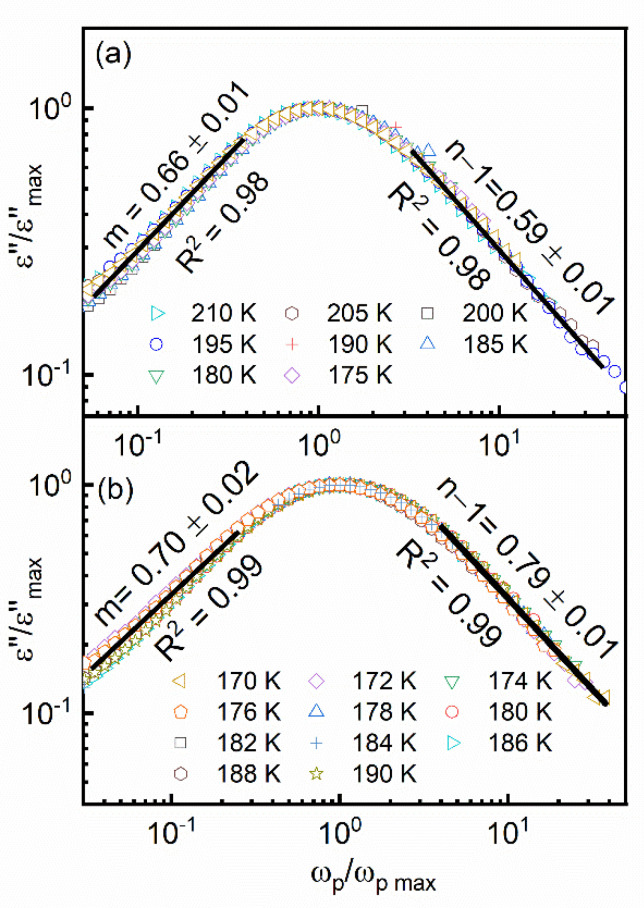
Normalized dielectric loss data obtained for DMAZnF (**a**) and DMAZnD (**b**) (log–log scale). Straight lines show linear fits to data points. For both the samples, the value of the high (*n* − 1) ‒and low-frequency (m) power−law exponent remained temperature invariant.

In Figure 7, normalized data representing the imaginary term of the complex permittivity are depicted. It can be observed that for both the studied samples, normalization resulted in a master curve exhibiting a single maxima peak. Moreover, it is clear from the graph that the dielectric response of both the investigated DMAZnF and DMAZnD followed the anomalous relaxation mechanism represented by the low- (m) and high-frequency (*n* − 1) power-law dependence of the imaginary part of dielectric permittivity on frequency, i.e.,:

(1) $$\varepsilon^{\prime \prime }\left(\omega \right)\propto {\left(\omega /\omega_{p}\right)}^{m}\;\;\;\;\;\mathrm{for}\;\omega <\omega_{p}$$

(2) $$\varepsilon^{\prime \prime }\left(\omega \right)\propto {\left(\omega /\omega_{p}\right)}^{n-1}\;\;\;\;\mathrm{for}\;\omega >\omega_{p},$$

where *ωp* = 1/*τ* denotes the loss peak frequency, τ is a characteristic relaxation time of the process, and 0 < *m*, *n* < 1. Furthermore, we found that within the entire investigated temperature range, in the case of the DMAZnF sample, the low- and high-frequency power-law exponents satisfied the relation m<n−1, whereas for the DMAZnD sample, the opposite relation, m≥n−1, was observed. It is known that relaxation data, for which *m* ≥ *n* − 1, can be interpreted within a relaxation scenario, leading to the well-known Havriliak–Negami (HN) relaxation function [36,37]:

(3) $$\varphi_{HN}^{*}\left(\omega \right)=\frac{1}{{\left[1+{\left(i\omega /\omega_{p}\right)}^{\alpha }\right]}^{\beta }},\;\;0<\alpha ,\beta <1$$

The HN function cannot be used to interpret the ‘less typical’ relaxation data, yielding the opposite inequality m<n−1. In this case, a relaxation scenario resulting in the Generalized Mittag–Leffler function (GML) should be applied [38]:

(4) $$\varphi_{GML}^{*}\left(\omega \right)=\frac{1}{{\left[1+{\left(i\omega /\omega_{p}\right)}^{-\alpha }\right]}^{\gamma }},\;\;0<\alpha ,\gamma <1$$

According to a theoretical model of the non-exponential two-power law relaxation response, a system responding either in the Havriliak–Negami or generalized Mittag-Leffler manner consists of a certain number of single dipolar relaxation contributions, which form a cluster with their local surroundings. In the case of the studied DMAZnF and DMAZnD, it is reasonable to assume that the DMA^+^ cation possessing dipolar moment may be treated as a single dipole-like object and that clusters were formed by the DMA^+^ cation and the neighboring framework atoms. The observed change in the measured relaxation response of the studied compounds after the framework deuteration may have been related to the fact that the framework stiffening may have ha d an impact on the DMA^+^ fitting into the crystal voids. This issue, however, requires further study and will be the subject of a separate analysis in the near future.

**Figure 8:**
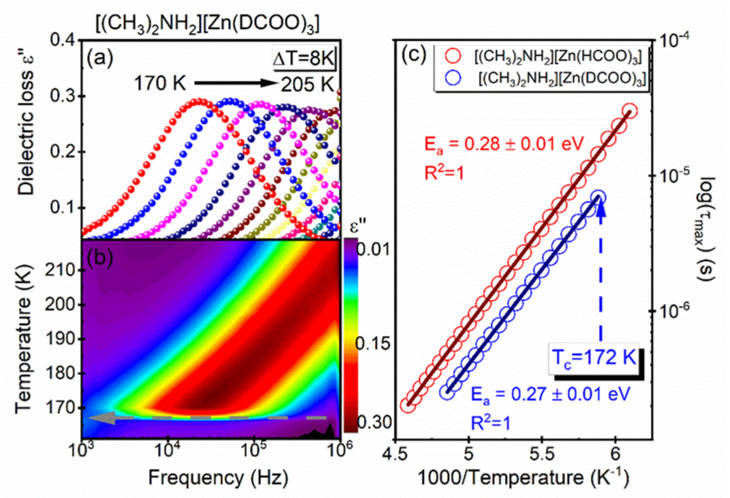
Frequency dependence of (**a**) the loss spectra, (**b**) temperature map and (**c**) relaxation map, with τ as a function of 1000/Temperature.

To determine the parameters of the relaxation dynamics of the observed dipolar process, the inverse temperature dependence of the mean dielectric relaxation time τ_max_ was analyzed as a function of the inverse of temperature (see Figure 8c). Both these dependencies exhibited linear behaviour, in accordance with the classical Arrhenius law, for which $\tau =Ae^{-\frac{E_{a}}{kT}}$ (where *E_a_* denotes the activation energy, A denotes the pre-exponential factor and k is the Boltzmann constant). The linear fit to the results produced an estimated activation energy equal to 0.27 eV for the DMAZnD (see Figure 8c).

It should be pointed out that the studied compound activation energy values did not change after the framework deuteration. A convergence of the activation energy values may have indicated that the mechanism of DMA^+^ cation movement in the HT phase was the same in the undeuterated sample and the sample with the deuterated framework. Some differences, however, may be observed between the relaxation times The shorter relaxation times coincided with the sample including a deuterated framework, i.e., the cation rotation was faster when the framework was stiffer. The above results may indicate a direct relationship between the stiffening of the framework and the DMA^+^ cation dynamics.

## 4. Conclusions

As a result of the investigations performed, it was found that the ((CH_3_)_2_NH_2_)(Zn(DCOO)_3_) (DMAZnD) framework deuteration had an impact on the physical properties and phase stability of the studied compound. It was shown that the replacement of hydrogen by deuterium atoms in the formate framework resulted in a phase transition temperature change. Namely, the DMAZnF underwent a reversible order–disorder phase transition at 156 K, while in the DMAZnD, the phase transition temperature rose to approximately 161 K. This observation indicates the contribution of the formate framework to the phase transition mechanism in the family of ammonium metal formates.

The powder diffractograms, Raman, and IR spectra of the compounds DMAZnD and DMAZnF proved that both the compounds are isomorphic. The analysis of the vibrational spectra suggests, however, that deuteration led to a slight increase in the HBs’ strength and the stiffening of the zinc-formate framework. As a result, the framework distortion at T_c_ was weaker for the DMAZnD compared to the DMAZnF. This in turn seems to affect the geometry and fit of the DMA^+^ cations in the crystal voids. As a result, the change in the CNC angle at T_c_ was larger for the DMAZnD than for the DMAZnF. However, the deuteration did not change the phase transition mechanism, as evidenced by the very similar temperature evolution of the bands related to the NH_2_ group. It is therefore concluded that the formate ion deuteration-induced increase in T_c_ was most likely caused by a disturbance in the delicate balance between the flexibility of the zinc-formate framework, the adaptability of the DMA^+^ cations to the voids, and the HBs’ strength.

The results of the dielectric spectroscopy measurements revealed the presence of a single-dipolar relaxation process in both the undeuterated and the deuterated sample. The relaxation times analysis, which allowed the estimation of the activation energy value, showed that the framework deuteration did not change the activation energy value of the DMA^+^ cations rotation. However, for both the studied samples, a non-exponential two-power law relaxation response was detected; the mutual relationship between the power law exponents suggested the Havrilak–Negami and Generalized Mittag–Lefler relaxation response of the DMAZnF and DMAZnD, respectively. It is reasonable to assume that the framework stiffening had an impact on the DMA^+^ cation’s ability to fit into the framework cavities and, in consequence, a change in the type of the dielectric response of the compound was observed. This issue, however, requires further study.

## Data Availability

The data presented in this study are available on request from the corresponding author.

## Supplementary Materials

The following are available online at https://www.mdpi.com/article/10.3390/ma14206150/s1. Figure S1: DSC data for DMAZnF and DMAZnD between 130 K and 280 K for cooling and heating run. Figure S2: The RT Raman and IR spectra of DMAZnD compared to DMAZnF. Figure S3: Thermal evolution of IR spectra measured for DMAZnD. Figure S4: A comparison of temperature dependencies of Raman and IR bands for DMAZnD and DMAZnF. Table S1: The wavenumbers along with intensity and proposed assignment of the observed IR and Raman bands at RT and at 80 K for DMAZnD.
