# Supplementary material for: Metal-Formate Framework Stiffening and Its Relevance to Phase Transition Mechanism
Source: Materials (Basel). 2021 Oct 16;14(20):6150. doi: 10.3390/ma14206150 (PMC8537347; doi:10.3390/ma14206150)
Supplement: Supplementary file 1 [file materials-14-06150-s001.zip › materials-1411328-supplementary.pdf]

Electronic Supporting information

for

Metal-formate framework stiffening and its relevance on phase transition mechanism

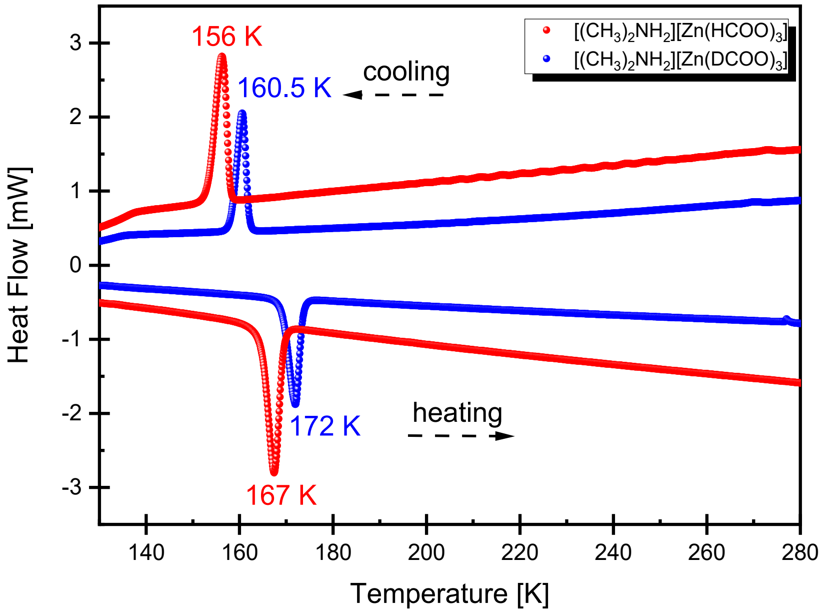

Figure S1 DSC data for ***DMAZnF*** *and* ***DMAZnD*** *between 130 K and 280 K for cooling and heating run.*

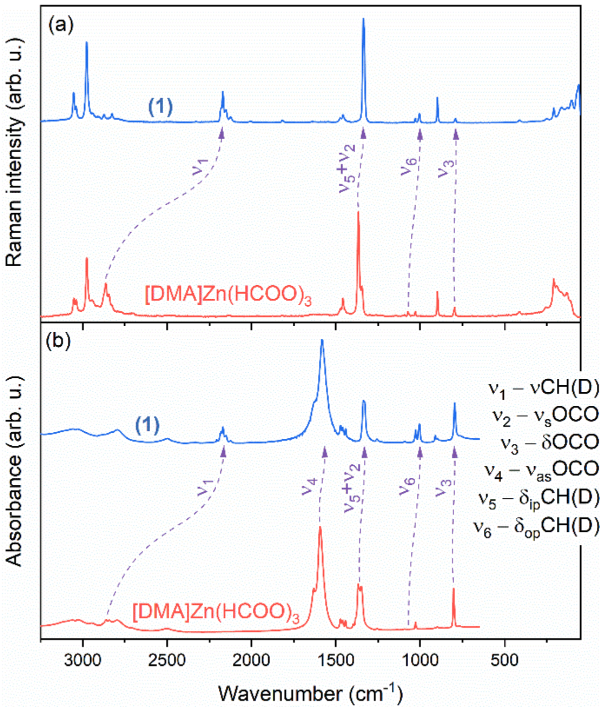

Figure S2 The RT Raman (a) and IR (b) spectra of ***DMAZnD*** *compared to* ***DMAZnF****.*

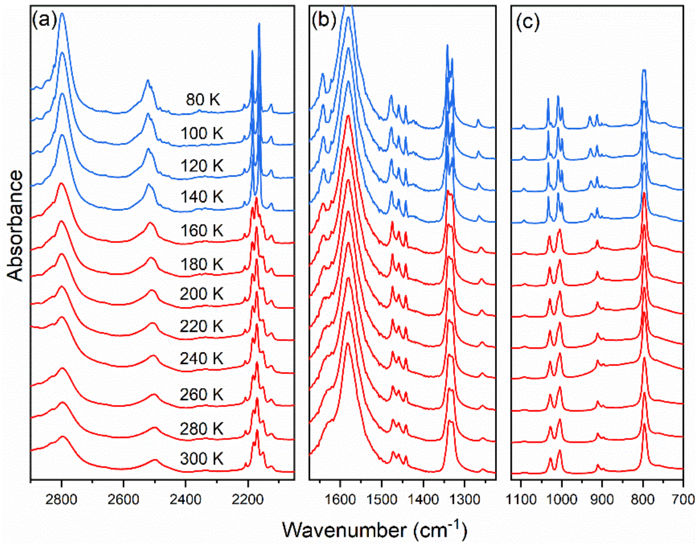

Figure S3 Thermal evolution of IR spectra measured for ***DMAZnD****.*

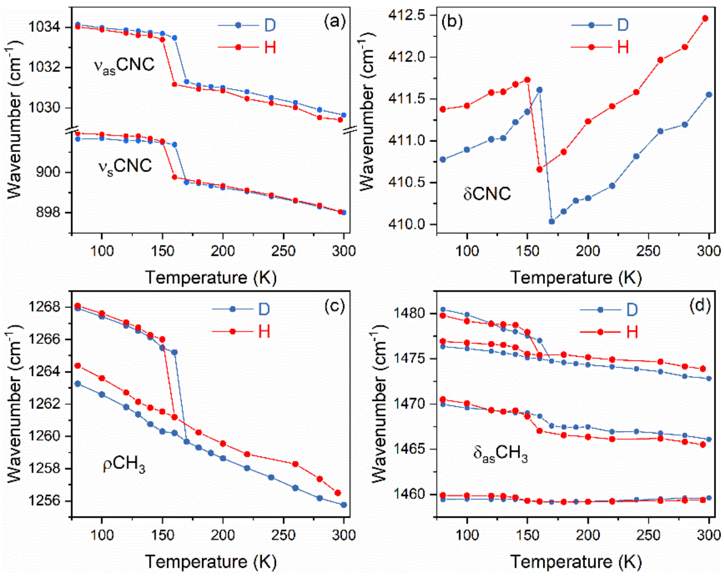

Figure S4 A comparison of temperature dependencies of Raman (a,b) and IR (c,d) bands, corresponding to ν_{as}CNC and ν_sCNC (a), δCNC (b), ρCH_3 (c), and $\delta_{as}CH_3$ (d), for ***DMAZnD*** *(D, blue) and* ***DMAZnF*** *(H, red)* [1]*; lines are guides for eyes.*

Tab. S1. The wavenumbers (cm^{-1}) along with intensity and proposed assignment of observed IR and Raman bands at RT and at 80 K for **DMAZnD** [a]

Raman		IR		Assignment
**RT**	**80 K**	**RT**	**80 K**	
		3108sh	3131w	overtone
3055m	3050s	3059m	3052w	$\nu_{as}CH_3$
3041w	3036m, 3033m	3032m		$\nu_{as}CH_3$
			3004w	νNH_2
2977s, 2943w	2974vs, 2948m		2975sh, 2952sh	$\nu_s CH_3$
2911w, 2876w, 2827w	2909vw, 2898vw, 2875w, 2834vw, 2824vw	2874sh, 2832sh	2926w, 2903w, 2879w, 2845w, 2824w	overtone
2795b	2787m	2794m	2800m	νNH_2
	2526vw	2500w, 2336vw	2551sh, 2523w, 2512vw, 2483vw, 2474vw, 2369vw, 2340vw	overtone
2180w, 2173m, 2151w, 2124w	2186m, 2161m, 2126w	2207w, 2181w, 2171m, 2152m, 2125w	2211w, 2186m, 2164m, 2125w	ν_1
2005vw, 1817w	2009vw, 1822w	2005vw	2009vw, 1855vw	overtone
1636vw	1640vw	1635s	1644m, 1622m	δNH_2

1587vw, 1547vw	1573vw, 1546vw	1579vs	1581vs	ν_4
1474w, 1458w	1482w, 1477sh, 1458w	1473m, 1466sh, 1459m	1481sh, 1476m, 1470sh, 1459m	$\delta_{as}CH_3$
	1442vw	1442m	1442m	$\omega NH_2+\delta_s CH_3$
	1432vw		1424m	$\tau NH_2+\delta_s CH_3$
1337vs	1341sh, 1338vs	1337vs	1349sh, 1342s	ν_2
1332sh	1332s, 1318vw	1330vs	1335s, 1331s	ν_5
1236vw, 1093vw	1232vw, 1094vw, 1054vw	1256w, 1092vw	1268w, 1263sh 1094w	ρCH_3
1030w	1034w	1028m	1033m, 1026w	$\nu_{as}CNC$
1006w	1009w, 999w	1100sh, 1005m	1009m, 999m	ν_6
	931vw, 915vw	906sh, 910w	930w, 913w	ρNH_2
898m	902m, 892vw	897vw	901w, 893vw	$\nu_s CNC$
796sh, 790w	797sh, 791w	796s, 761vw	800s, 798s, 795s	ν_3
412w	411w			δCNC
	334w			τCH_3
249w, 209w, 164w, 129w, 102m, 69sh, 59m	267w, 253vw, 225vw, 210m, 196w, 175m, 166sh, 157m, 139sh, 134m, 129sh, 120sh, 112sh, 108m, 104sh, 94w, 79m, 63w, 54m			lattice modes

[a]Key: vs, very strong; s, strong; m, medium; w, weak; vw, very weak; sh, shoulder; s, symmetric; as, antisymmetric; ν, stretching; δ, bending (scissoring); ρ, rocking; τ, twisting; ω, wagging; ν_1, CH (CD) stretching; ν_2, symmetric OCO stretching; ν_3, OCO bending; ν_4, antisymmetric OCO stretching; ν_5, in-plane CH (CD) bending; ν_6, out-of-plane CH (CD) bending

References

[1] Mączka M, Ptak M and Macalik L 2014 Infrared and Raman studies of phase transitions in metal–organic frameworks of [(CH_3)$_2$$NH_2$][M($HCOO$)$_3$] with M=Zn, Fe *Vib. Spectrosc.* **71** 98–104
